# New Challenges for the Design of High Value Plant Products: Stabilization of Anthocyanins in Plant Vacuoles

**DOI:** 10.3389/fpls.2016.00153

**Published:** 2016-02-16

**Authors:** Valentina Passeri, Ronald Koes, Francesca M. Quattrocchio

**Affiliations:** Plant Development and (Epi)Genetics, Swammerdam Institute of Life Sciences, University of AmsterdamAmsterdam, Netherlands

**Keywords:** Anthocyanin, fading, product stabilization, health-promoting products, vacuole

## Abstract

In the last decade plant biotechnologists and breeders have made several attempt to improve the antioxidant content of plant-derived food. Most efforts concentrated on increasing the synthesis of antioxidants, in particular anthocyanins, by inducing the transcription of genes encoding the synthesizing enzymes. We present here an overview of economically interesting plant species, both food crops and ornamentals, in which anthocyanin content was improved by traditional breeding or transgenesis. Old genetic studies in petunia and more recent biochemical work in brunfelsia, have shown that after synthesis and compartmentalization in the vacuole, anthocyanins need to be stabilized to preserve the color of the plant tissue over time. The final yield of antioxidant molecules is the result of the balance between synthesis and degradation. Therefore the understanding of the mechanism that determine molecule stabilization in the vacuolar lumen is the next step that needs to be taken to further improve the anthocyanin content in food. In several species a phenomenon known as fading is responsible for the disappearance of pigmentation which in some case can be nearly complete. We discuss the present knowledge about the genetic and biochemical factors involved in pigment preservation/destabilization in plant cells. The improvement of our understanding of the fading process will supply new tools for both biotechnological approaches and marker-assisted breeding.

## Introduction

**FIGURE 1:**
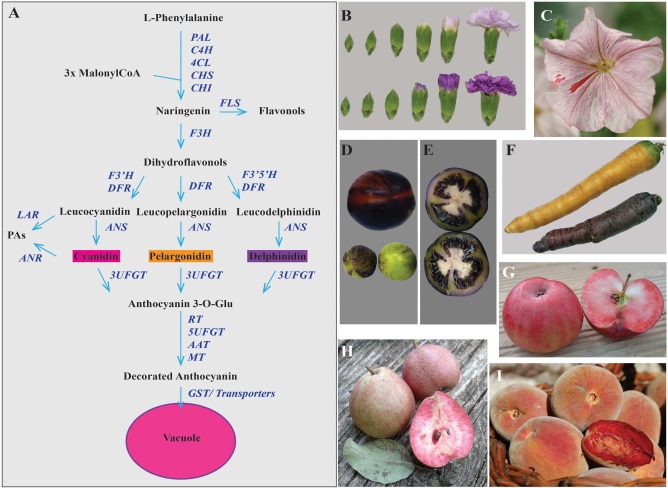
**Anthocyanin accumulation in different plant products.** **(A)** Scheme of the biosynthetic pathway for different flavonoid pigments among which anthocyanins. The main *enzymes* catalyzing the reactions in the pathway are reported in blue. *PAL, phenylalanine ammonia-lyase; C4H, cinnamate 4-hydroxylase; 4CL, 4-coumarate-CoA ligase; CHS, chalcone synthase; CHI, chalcone isomerase; FLS, flavonol synthase; F3H, flavonoid 3 hydroxylase; F3′H, flavonol 3′ hydroxylase; F3′5′H, flavonol 3′,5′ hydroxylase; DFR, dihydroflavonol 4-reductase; LAR, leucoanthocyanidin reductase; ANR, anthocyanidin reductase; ANS, anthocyanidin synthase; 3UFGT, UDP glucose:flavonoid 3-*O*-glucosyltransferase; RT, rhamnosylation at three; 5UFGT: glucose:flavonoid 5-*O*-glucosyltransferase; AAT, anthocyanin acyltransferase; MT, methyltransferase; GST, glutathione *S*-transferase.* PAs, proanthocyanidins. In **(B)** Moondust (up) and Moonshadow (down) transgenic carnations produced by Florigene/Suntory; **(C)** Petunia *ph6* unstable mutant (transposon insertion) in the hybrid W138xR153 background. The red spots and sectors are due to *PH6* reversion. In **(D)** transgenic tomato fruits from plants expressing the *35S:SlANT1* construct. Immature green and red ripe fruits with anthocyanin-rich sectors in the peel; **(E)** green tomatoes from the same plants as in **(D)** showing purple flesh, locular cavities and seeds. **(F)** Orange and purple carrots. In **(G)**, **(H),** and **(I)** ancient varieties of *Rosaceae* species with anthocyanin-rich flesh. These fruits are locally known as: **(G)** “mela rossa dentro incarnato” (apple variety), **(H)** “pera cocomerina” (pear variety) and **(I)** “pesca sanguinella” (peach variety).

Anthocyanins are flavonoid pigments conferring red, blue and purple colors to plant tissues. Because they are visible to the naked eye, these pigments are a model for genetics, molecular biology and cell biology. Consequently, both structural and regulatory genes of the biosynthetic pathway are identified in a plethora of species (**Figure 1A**). A complex of highly conserved WD40, bHLH and MYB proteins (MBW complex) activates the transcription of structural genes encoding enzymes of the anthocyanin pathway (Koes et al., 2005; Jaakola, 2013). In all species analyzed, the WD40 is expressed ubiquitously, whereas expression of bHLH and MYB factors is confined to pigmented tissues. The bHLH regulators hook up with the WD40 partner to activate downstream genes involved in multiple pathways like anthocyanin and tannin production, vacuolar acidification and cell shape, through interactions with different MYB proteins, which are main determinants of the specificity of the complex (Koes et al., 2005; Ramsay and Glover, 2005). The MYB component of the MBW complex that activate the (pro)anthocyanin pathway is able to activate transcription of its bHLH partner and is therefore consider a “master regulator” as it can, alone, induce activation of the pathway (Spelt et al., 2000; Nesi et al., 2001; Kiferle et al., 2015).After synthesis, anthocyanins are transported to the vacuolar lumen where they are stored. This process is studied by several groups (Francisco et al., 2013; Chanoca et al., 2015; Hu et al., 2016) but it is still not fully understood in spite of the substantial role it might play in the final anthocyanin content in plant tissues.

Plant products rich in anthocyanin like berries, eggplant, grape, and red cabbage, are part of the human diet. Several studies reported that anthocyanin-intake prevents the onset and development of degenerative diseases. Some example of the health promoting effects of anthocyanins are stimulation of visual acuity and reduction of retinal damage (Kalt et al., 2014; Giampieri et al., 2015; Wang et al., 2015), decreased expression of inflammatory biomarkers (Samadi et al., 2015), diminished risk of type-2 diabetes mellitus (Guo and Ling, 2015), reduced weight gain (Titta et al., 2010) and anti-cancerogenic activity (Butelli et al., 2008; Forbes-Hernandez et al., 2015; Vlachojannis et al., 2015). By *in vitro* simulation of the gastrointestinal system and animal and human tests, anthocyanins were shown to remain bio-accessible during digestion (Kalt et al., 2014; Oliveira and Pintado, 2015; Olejnik et al., 2016).

The presence of anthocyanin in plant tissues positively affects their market value in addition by increasing the aesthetical appeal and by reducing softening, shriveling, rotting and fungal infection (Zhang et al., 2015c). Furthermore color novelty is a major driving force in the ornamentals and cut flower industry.

Increased anthocyanin content is, for all mentioned reasons, an obvious goal for crop breeding and biotechnology. Therefore combinations of classical and molecular methods, have been used to generate new varieties with enhanced anthocyanin content as well as different colors and pigmentation patterns.

Till now, research in ornamental and food crops aimed to alter genes controlling anthocyanin synthesis, since it was taken for granted that the end products are stable once they are deposited in the vacuole. However, for fruits, flowers and leaves of several species it is known that anthocyanin may disappear again during development in a regulated manner that depends, for example on environmental conditions (Oren-Shamir, 2009).

Here we review the state of the art in improving anthocyanin production in plant tissues and report recent insights into the (in)stability of anthocyanins in vacuoles, suggesting that the understanding of the mechanism behind anthocyanin stabilization *in planta* is required for breeding and biotechnology to take the next step toward plant varieties with increased economical and nutraceutical value.

## Studying Flower Pigmentation Taught us How to Color Our Food

Much of the current knowledge on anthocyanin chemistry and genetics originates from studies on flower pigmentation in model species. Some of the results have been applied to generate new varieties of cut flowers and ornamental flowering plants with novel colors and pigmentation patterns.

The substrate specificity of the enzymes of the anthocyanin pathway determines the final pattern of chemical decorations and thereby the pigment color (Provenzano et al., 2014; Rinaldo et al., 2015). Together with the understanding of the biosynthetic pathway regulation (Koes et al., 2005; Jaakola, 2013), this knowledge was applied to enhance the nutraceutical value and the appeal of several economically relevant plant products.

Traditional breeding has produced an array of colors in different species but the top-selling cut flowers rose, chrysanthemum, carnation and lily do not have blue in their pallet, while petunia lacks red/orange (Holton and Tanaka, 1994; Forkmann and Heller, 1999). New colors were obtained changing the decoration pattern on the basic skeleton of anthocyanins (**Figure 1A**) in roses, chrysanthemum and carnations. The expression of an exogenous *flavonol 3′,5′ hydroxylase* (*F3′5′H*) combined with an heterologous *dihydroflavonol 4-reductase* (*DFR*) accepting a three-hydroxylated substrate, leads to accumulation of delphinidin (Katsumoto et al., 2007; Tanaka et al., 2008) and to lilac and purple flowers in rose and carnation (**Figure 1B**). Orange and red colors from pelargonidin-based anthocyanins were obtained in petunia by suppressing the *flavonoid hydroxylases F3′H* and *F3′5′H*, and expressing a *DFR* with specificity for mono-hydroxylated substrates (Meyer et al., 1987). New colors are also obtained by changing the anthocyanin pattern of methylation, glycosylation, and acylation (Provenzano et al., 2014; Du et al., 2015; Morita et al., 2015).

The dynamics of metabolic flows affects channeling of precursors toward anthocyanin production (Zvi et al., 2012; Sheehan et al., 2015; Zhang et al., 2015b) and this should be considered when designing strategies to generate genotypes with new colors or enhanced anthocyanin content.

Flower pigmentation patterns originate from differential expression of the structural genes in different cells. While irregular patterns are mostly due to transposon insertions in structural and/or regulatory genes (**Figure 1C**; Lister et al., 1993; Spelt et al., 2000; Itoh et al., 2002), flecks, sector veins and coloration of different flower parts are due to differential expression of genes encoding for MYB proteins of the MBW transcription complex regulating the anthocyanin pathway.

In the genus *Antirrhinum* variation in activity of the MYB genes *Rosea* and *Venosa* regulates pigmentation in different flower parts (Stracke et al., 2001) and in petunia, different members of the same clade of MYB regulators independently pigment petals, anthers and tube (Tornielli et al., 2009). Similarly, in *Phalaenopsis* orchids three MYBs control spotting and venation patterns by activation of structural genes expression in the sepals/petals (Hsu et al., 2015). Ectopic expression of the *Arabidopsis* anthocyanin MYB regulator *PAP1* in roses results in enhanced pigmentation in leaves and flowers (Zvi et al., 2012).

From the observation of how pigmentation patterns diverged during evolution we learned that MYB regulators of anthocyanin biosynthesis are the best tool to alter anthocyanin production without affecting other processes. This is because their bHLH and WDR partners are involved in several other processes and changes in their activity would either be insufficient or have pleiotropic effects. Factors affecting pigment production more indirectly, like hormones, sugar concentration (Loreti et al., 2008; Zhou et al., 2009) or high light and cold (Lotkowska et al., 2015; Zhang et al., 2015a), usually have dramatic side effects on the plant physiology.

The picture of anthocyanin synthesis and regulation gained from studies in flowers was confirmed in several crops where homolog MBW complexes regulate pigment accumulation in different plant parts.

Modern crops are the result of a domestication process that, for most species, went on for the last 10.000 years. Selection resulted sometimes in the loss of pigmentation in some plant parts. Pigmentation in tomato fruits, for example, was probably a trait indirectly counter-selected by breeding as the fruits of several closely related wild *Solanum* species are colored. The introgression in domesticated tomato of two loci, *Aft* (*Anthocyanin fruit*) and *atv* (*atroviolacea*) from wild *Solanum*, results in the accumulation of anthocyanins in the epidermis and the pericarp of the fruit (Povero et al., 2011), indicating that it is possible to restore fruit pigmentation by adding few genes. In fact, ectopic expression of any of the R2R3-MYB genes *SlAN2* and *SlANT1* (Kiferle et al., 2015) is sufficient to get purple tomatoes (**Figures 1D,E**). As SlAN2 and SlANT1 proteins activate the whole biosynthetic pathway and stress can activate *SlAN2* transcription, lack of pigmentation in cultivated tomato fruits is not due to mutations in enzyme encoding genes or to loss of function of one of the two MYBs. Rather, changes in the regulation of the MYBs, resulted in inactivity in fruits. Expression of *DELILA* and *ROSEA1* (respectively a bHLH and a MYB) from snapdragon result in intensely purple tomatoes fruits which have health-promoting effects in a mouse model (Butelli et al., 2008). High expression of a different type of MYB (*MYB12*) in tomato stimulates the production of complex mixtures of flavonoids, by reprogramming primary metabolism toward the production of substrates for the phenylpropanoid pathway. The combination of *MYB12* and transcription factors specific for the anthocyanin pathway further boosts anthocyanin production (Zhang et al., 2015c).

MYB genes are also responsible for pigmentation also in grape berries (Kobayashi et al., 2002; Walker et al., 2007), blood oranges (Butelli et al., 2012), apples and pears (Takos et al., 2006; Ban et al., 2007; Yuan et al., 2014). Some apple genotypes show red flesh and share a single ancestor, the *Malus sieversii f. niedzwetzkyana* wild apple native of Central Asia (Harris et al., 2002). The expression pattern of *MdMYB10* in red flesh apples correlates with anthocyanin gene expression (Espley et al., 2007), and a minisatellite-like structure in its promoter increases *MdMYB10* transcription and the accumulation of anthocyanin in leaves, flowers, and fruit cortex (Espley et al., 2009). Max Red Bartlett, a red-skinned European pear variety, gives occasionally green-skinned fruits in which *PcMYB10* expression is silenced due to the methylation of two regions in its promoter (Wang et al., 2013).

The purple cauliflower (*Brassica oleracea* var. *botrytis*) originates from a spontaneous mutant found in a cauliflower field over 20 years ago (Chiu et al., 2010). This mutation results in up-regulation of transcription of the *Pr* gene encoding for a MYB. Purple varieties are also known for carrots (**Figure 1F**), onions and potato (De Jong et al., 2004). Several more examples could be added to this list, showing that MYBs are indeed “master regulators” of anthocyanin biosynthesis and their expression pattern determines pigmentation patterns in plants.

The market request of high anthocyanin content food, led to the rediscovery of pigment-rich varieties, which were nearly forgotten. These ancient varieties of apples, pears and peaches (**Figures 1G–I**) are still poorly studied, but are a priceless source of interesting alleles to be introduced into market varieties.

Selection in agriculture probably favors mutations in MYB genes, over mutations in their bHLH and WD40 partners or in structural genes, because they are the least pleiotropic and because gain of function mutations are more likely to activate anthocyanin synthesis in new tissues. Strategies for improving anthocyanin production in crops by both breeding and genetic engineering mimics natural selection, acting on MYBs to tune the expression of anthocyanin structural genes.

## Higher Production Not Always Means Higher Yield, At Least for Anthocyanins

There are now sufficient tools to improve pigment production and color displayed by fruits and flowers. However, we have little understanding of the role played by degradation of anthocyanins on the total yield in fruits and on color in flowers.

It is often taken for granted that anthocyanins, once accumulated in the vacuole, are stable. However, few studies describe anthocyanin turn over and addressed whether this is due to enzymatic activity, spontaneous reactions or a combination of both (Oren-Shamir, 2009).

Color fading is reported for several species and here we briefly summarize illustrative examples reported in literature and/or known from everyday life.

**FIGURE 2:**
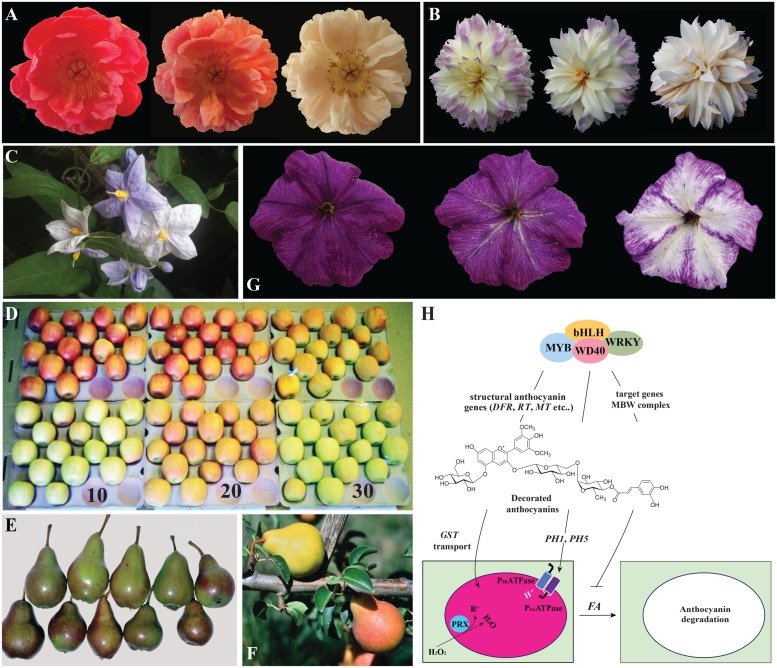
**Anthocyanin degradation in plants.** **(A)** The same peony flower photographed different days (between the first and the last picture are 6 days) after opening. In **(B)** the same dahlia flower photographed at different days (between the first and the last pictures are about 2 weeks). **(C)** *Solamun wrightii* flowers photographed on the plant. The young buds and just open flowers are intensely pigmented, while older flowers are totally white indicating strong fading of the anthocyanin pigments. **(D)** Color change in red (upper row) and green (bottom row) ‘Cripps Pink’ apples exposed to moderate light at 10, 20, and 30°C for 6 days. The green apples accumulated anthocyanin at 20°C while the red apples loose anthocyanin at 30°C (Steyn et al., 2004). **(E)** Bleaching of red color (upper row) at the sun-exposed side of Rosemarie fruits compared to fruits receiving less intense light (bottom row) which maintain more intense pigmentation. **(F)** Rosemarie pears: one fruit has lost its red color and turned yellow on the tree. This phenomenon is reported for Rosemarie pears fruits that bent over during development resulting in pinching of the peduncle. **(G)** Petunia flower photographed at different moments after opening and showing strong color fading. This is a *ph4* mutant line in a *FADING* background accumulating malvidin. **(H)** Scheme summarizing our present understanding of color fading in plant cells. Similar transcription factor complexes consisting of MYB, bHLH, WD40 and WRKY factors control anthocyanin biosynthesis (through the transcription of the structural genes encoding for the enzymes of the pathway) and vacuolar acidification (through the transcription of the two pumps PH1 and PH5). Anthocyanin are sequestered to the vacuolar lumen. When the anthocyanin molecules are highly decorated and a dominant allele of the *FADING* (*FA*) gene is present, color fading takes place as consequence of anthocyanin degradation, probably in the vacuolar lumen. This mechanism is blocked by the activity of the MBW complex indicating that target genes of these transcription factors might protect anthocyanins from the effect of *FA*.

In some plants, anthocyanins protect the photosynthetic apparatus from light damage in young leaves, and are lost later in development, enabling more light to enter the tissues (Steyn et al., 2002, 2004; Nissim-Levi et al., 2003). Instead, apple and pear peels show changes in pigmentation in response to temperature and/or light (**Figures 2D–F**; Steyn et al., 2004, 2009). In blood oranges, on the other end, anthocyanin content reaches a maximum in the fully ripe fruit, to decreases at latter stages when β-D-Glucosidase activity increases giving the formation of aglycons which are possible substrates for degradation by polyphenol oxidase, abundant in these fruits (Barbagallo et al., 2007). Polyphenol oxidases are also suspected to induce fading together with peroxidases in litchi fruits (Reichel et al., 2011) where an anthocyanin degradation enzyme (ADE) was identified as vacuolar laccase secreted to the extracellular space at pericarp browning (Fang et al., 2015).

Flowers turned out to be an excellent model to study color fading, which is observed for instance, in peony (**Figure 2A**), *Hibiscus*, orchids (Burg and Dijkman, 1967; Zhao et al., 2012; Shimokawa et al., 2015), dahlias (**Figure 2B**) and several *Solanum* species (**Figure 2C**). In commercial varieties of flowers, fading strongly affects the market value. One of these is aster, where the inhibition of color fading by magnesium is suggested to come from the formation of pigment-metal complexes (Shaked-Sachray et al., 2002). Although similar results were reported for grape cell suspensions (Sinilal et al., 2011), there is no direct evidence for the presence of metalloanthocyanin in these species.

Also the petals of *Brunfelsia calycina*, a *Solanaceae* shrub, fade from blue to complete white within few days after flower opening (Vaknin et al., 2005). Protein and mRNA synthesis inhibitors prevent anthocyanin degradation in these petals suggesting that fading is an active process. Interestingly, cytokinin treatment delays petal senescence but not anthocyanin degradation, suggesting that fading is independent from petal senescence and the accompanying increase in pH. Peroxidase activity correlates in time with anthocyanin degradation and recently, Zipor et al. (2015) characterized a candidate vacuolar peroxidase, *BcPrx01*, which transcript and protein level increase during fading. Furthermore, total protein extracts from brunfelsia petals induce *in vitro* fading of anthocyanins with different decorations extracted from petunia petals after addition of H_2_O_2_, suggesting a not substrate-specific mechanism. However, direct evidence that this *in vivo* reaction mimics the degradation seen *in vivo* is currently lacking.

The color of anthocyanins is affected by the pH of the vacuolar lumen where they accumulate. A strongly acidic lumen results in red, and a less acidic one in blue. In *Petunia*, blue flowering mutants define the loci *PH1* to *PH7* (Koes et al., 2005) which control vacuolar acidification in petals. *PH1* and *PH5* encode a heteromeric proton pump, transcriptionally controlled by the *AN1-PH4-AN11-PH3* complex (a bHLH, a MYB, a WDR and a WRKY transcription factors) sharing components with the MBW complex regulating anthocyanin biosynthesis. Thus, pigment synthesis and vacuolar acidification are controlled by the same regulatory network (Verweij et al., 2008; Faraco et al., 2014). In petunia *ph3*, *ph4* and *ph6* mutants that contain the dominant allele of the *FADING* (*FA*) locus (de Vlaming et al., 1982, 1983), nearly complete degradation of anthocyanin occurs after flower opening (**Figure 2G**). This process is restricted to the flower limb, while flower tube and pollen maintain their full color. Color fading is in petunia much stronger for highly substituted anthocyanins, such as 3-rutinosido(p-coumaroyl)-5glucoside anthocyanins, whereas 3-glucosides and 3-rutinosides only weakly fade and anthocyanin methylation has no effect (de Vlaming et al., 1982). As reported above, *in vitro*, brunfelsia protein extracts equally destabilize differently substituted anthocyanins from petunia (Zipor et al., 2015). This discrepancy might have different explanations: (i) the *FA* gene product has specificity for highly substituted anthocyanin molecules and the *in vitro* reaction does not reflect the one *in vivo*, (ii) the specificity of the fading mechanism in brunfelsia is different from the one in petunia, or (iii) FA activity is dependent on genes that genetically linked with genes determining the anthocyanin sunstituion patter, such as *Rhamnosyl Transferase* (*RT*) and *Glucosylation at Five* (*GF*; Quattrocchio et al., 2006).

Our limited understanding of the mechanism of anthocyanin fading, coming from experiments in brunfelsia and genetic analysis in petunia, is summarized in **Figure 2H.** Decorated anthocyanin molecules are synthesized under the control of the MBW transcription complex and transported to the vacuolar lumen where their color is affected by the pH of the environment. This is determined by the PH1/PH5 pump which expression is also regulated by the MBW complex. In the vacuole, peroxidases modulate the concentration of free radicals and water peroxide, which can affect anthocyanin stability. Under these conditions anthocyanins are relatively stable (also in the presence of the FA allele), as compared to mutants for the MBW complex which are depleted in expression of all its target genes.

On the contrary of what suggested elsewhere (Oren-Shamir, 2009), fading in petunia is not merely a change in color due to high vacuolar pH in mutants. Mutations in *PH1* and *PH5* increase vacuolar pH in the same extent than mutations in the MBW complex, but are not accompanied by color loss (Verweij et al., 2008; Faraco et al., 2014). Fading in *ph4*, *ph3*, and *ph6* must therefore be due to down-regulation of other target genes of the MBW complex (Quattrocchio et al., 2006) in combination with the presence of a dominant *FADING* allele (de Vlaming et al., 1982).

The MBW complex controls several genes encoding enzymes of the anthocyanin pathway (Quattrocchio et al., 1998; Spelt et al., 2000), PH1 and PH5 (Verweij et al., 2008; Faraco et al., 2014) and at least 10 others of unknown function (Verweij et al., 2008). Which of these genes protect anthocyanins from the action of *FADING* can only be speculated. Their characterization via loss and gain of function study will shed light on this point, and will unravel which cellular mechanism protects anthocyanins from massive degradation.

The occurrence of fading obviously affects the final yield of anthocyanins diminishing the effect of synthesis improvement achieved by breeding or transgenesis (e.g., by modulation of the expression of MYB regulators). For this reason, the identification of the factors controlling fading of pigments as well as its inhibition, opens possibilities of further improvement of the content of these compounds in the final plant products.

## Conclusion

Anthocyanin-rich plants produced by traditional breeding or biotechnology, could contribute to human health reducing the incidence of major diseases (Martin et al., 2011), while new flower colors and patterns (Yoshida et al., 2009; Tanaka and Brugliera, 2013; Zhao and Tao, 2015) are interesting for the ornamental market. Success was booked in producing plants with enhanced anthocyanin synthesis by increasing the expression of MYB factors that activate transcription of structural anthocyanin genes. However, degradation also contributes to the final anthocyanin yield in plant products making the understanding of this phenomenon important for future strategies of crop improvement.

Studies in brunfelsia provide insight into the biochemistry of anthocyanin degradation (Zipor et al., 2015).

It is unclear whether a certain degree of anthocyanin degradation, is functional to the plant. So far only speculations are possible. Anthocyanins protect tissues from free radicals and in some species accumulate in seedlings where they shield the photosynthetic machinery from light. Their degradation later in development probably improves photosynthesis (Gould et al., 2002b). In brunfelsia, anthocyanin degradation in flowers is accompanied by release of fragrant volatiles and both processes could be signals for pollinators (Zipor et al., 2015). However, no evidence is available for correlations between the two phenomena. Reactive oxygen species (ROS) formed in aging flowers or maturing fruits from photo-oxidation, photorespiration, and Mehler reaction, could induce anthocyanin degradation and this might protect other cellular components from damages (Hernández et al., 2009). Moreover anthocyanins inhibit Fenton hydroxyl radical generation by scavenging superoxide and hydrogen peroxide (Gould et al., 2002a, 2010). A better characterization of the genes/factors involved in color fading will answer to the many questions we presented here and open the possibility to ‘design’ plant cells with stable vacuolar content. Mutants makes it possible to approach the characterization of the FADING locus and of the MBW target genes involved in anthocyanin stabilization. Considering that anthocyanins are not stable outside the vacuole (Mueller et al., 2000), the MBW complex could control vacuolar physiology and mutants might have vacuolar defect resulting in anthocyanin leakage. Factors involved in both fading and its prevention could function in totally unrelated pathways. Their participation in massive anthocyanin degradation might be a peculiarity of rare genotypes that amplify a moderate pigment loss normally occurring after vacuolar accumulation.

Genetic analyses in species, like petunia, where well-defined mutants affecting this phenomenon are available (de Vlaming et al., 1982, 1983; Quattrocchio et al., 2006) open the way to identify the genes that determine anthocyanin trun-over *in vivo*, to assess whether complete disappearance of color is an “accident” originating from human selection during crop domestication, and to gain tools to improve stabilization of anthocyanin (and possibly also other products) in the vacuolar lumen.

## Author Contributions

VP has searched the literature, collected the photographic information and written the manuscript. FQ and RK have conceived the idea and helped with writing the manuscript.

## Conflict of Interest Statement

The authors declare that the research was conducted in the absence of any commercial or financial relationships that could be construed as a potential conflict of interest.
